# Biochemical Constituents and in Vitro Antioxidant and Anticholinesterase Potential of Seeds from Native Korean Persimmon Genotypes

**DOI:** 10.3390/molecules21070893

**Published:** 2016-07-08

**Authors:** Saqib Bilal, Abdul Latif Khan, Muhammad Waqas, Raheem Shahzad, Il-Doo Kim, In-Jung Lee, Dong-Hyun Shin

**Affiliations:** 1 School of Applied Biosciences, Kyungpook National University, Daegu 41566, Korea; saqib043@yahoo.com (S.B.); agronomist89@gmail.com (M.W.); raheemshehzad@ymail.com (R.S.); ijlee@knu.ac.kr (I.-J.L.); 2 UoN Chair of Oman’s Medicinal Plants & Marine Natural Products, University of Nizwa, Nizwa 616, Oman; latifepm78@yahoo.co.uk; 3 Department of Agriculture, Abdul Wali Khan University Mardan, Mardan 23200, Pakistan; 4 International Institute of Agriculture Research & Development, Kyungpook National University, Daegu 41566, Korea; ildookim@hanmail.net

**Keywords:** reactive oxygen species, Alzheimer’s disease, phenolic content, amino acids, fatty acids, human health

## Abstract

In the current study, the functional and biochemical potential of the seeds of four persimmon cultivars (PC1, PC2, PC3 and PC4) and their role against oxidative stress and acetylcholinesterase (AChE) inhibition were evaluated. In terms of biochemical compositions, free amino acids, fatty acids and organic acids analysis was performed. The free amino acids ranged from 2617.31 (PC2) to 3773.01 μg∙g^−1^ dry weight (PC4). Oleic acid and linoleic acid were the principal fatty acids, which were significantly higher in PC4 and PC1, respectively. PC4 presented the highest amount of organic acid content (4212 mg∙kg^−1^), whereas PC2 presented the lowest (2498 mg∙kg^−1^). PC2 contained higher total phenolic content and flavonoid content, whereas PC3 had the lowest amount as compared to other cultivars. The in vitro DPPH, ABTS and superoxide anion radicals scavenging activity increased in a dose-dependent manner, whereas PC2 showed significantly higher scavenging activities as compared to PC1, PC2 and PC4 types. In the case of AChE inhibition, PC4 showed a moderate activity (67.34% ± 1.8%). In conclusion, the current findings reveal that the studied persimmon seeds cultivars are a source of bioactive natural antioxidants and AChE inhibitors. Such natural products could be employed in pharmaceutical and food industries, whilst can also be considered for the treatment of neurodegenerative diseases such as Alzheimer’s.

## 1. Introduction

Oxidative stress, caused by an imbalance of the antioxidative defense mechanisms and the production of reactive oxygen species (ROS), is considered a key agent of oxidative damages such as cancer, Alzheimer’s, diabetes and aging [1]. ROS are highly reactive molecules containing an unpaired electron which are primarily produced by oxidation during biological reactions or exogenous factors [2]. In the human body, various ROS such as the superoxide anion radical, the hydroxyl radical and hydrogen peroxide are produced by different exogenous and endogenous sources and factors. Exogenous sources of ROS include tobacco smoke, certain pollutants, organic solvents, chlorinated compounds, ions radiations and pesticides [3]. Mitochondria, peroxisomes, cytochrome P450 metabolism and inflammatory cell activation appear to be the major endogenous sources of ROS produced by cells [4]. The overproduction of free radicals or ROS may favor many chronic and degenerative damages in the human body such as the induction of aging, heart diseases, cancer, Alzheimer’s disease and other various abnormal physiological functions [5]. A specific amount of exogenous antioxidants is frequently required to counteract ROS.

Recently, many studies have recommended dietary antioxidant consumption to prevent from ROS induced damages. Polyphenol-rich foods, fresh fruits, vegetables and teas have potential protective effects against certain diseases. Their protection effect against oxidative damage is mainly attributed to the presence of significant amount of antioxidants such as flavonoids, phenolic acids, vitamins, anthocyanins and other phenolic compounds [6]. These natural antioxidants have the capability of terminating the free radical chain reaction by safely interacting with free radicals before any damaged is caused to body [7]. Besides their potential beneficial effects, natural antioxidants are of great interest in the pharmaceutical, cosmetic and in particular, the food industries as replacements for synthetic antioxidants [8], because they more effectively retard the peroxidation process [9]. Several synthetic antioxidants are used to inhibit lipid peroxidation in foodstuffs, such as butylated hydroxytoulene (BHT), butylated hydroxyanisole (BHA), tert-butyl hydroquinone (TBHQ) and gallate (PG), but previous studies suggest that synthetic antioxidants may be quite unsafe and deleterious to human health, resulting in carcinogenesis and liver damage [10].

In recent years, the use of non-toxic natural antioxidants available in foods such as vegetables and other biological substances has attracted considerable attention due to their significant safety, nutritional and medicinal value [11]. Natural antioxidants contain abundant amounts of phenolic compounds such as phenolic and flavonoid acids, tocotrienols, stilbenes, carotenoids and ascorbic acid. The beneficial effects of natural antioxidants are ascribed mainly to their high levels of phenolic compounds [7,12]. Most of the phenolic compounds available in foods are phenolic acids and flavonoids. The nutritional value of fruits for the characterization of possible beneficial effect against the oxidative stresses due the presence of biologically active compounds such as organic acids, fatty acids and amino acids has been widely discussed [13]. The nutritional and pharmacological role of amino acids is well known, and particularly the role of essential amino acids in peptide formation whilst exhibiting antioxidant activity have been emphasized extensively. Various saturated and unsaturated fatty acids such as oleic and linoleic acid have widely been recognized for their action in lowering the risk of ROS-induced diseases, i.e., tumors, inflammatory and heart diseases, and for enhancing the immune system [14]. The presence of different organic acids has been regarded essential to the organoleptic properties of fruits, and these acids can possess vital roles in human health by counteracting ROS-induced oxidative damages as well. Organic acids have also been recognized to be acidulant agents [15]. Epidemiological studies have demonstrated that the consumption of fruits is linked to reduced risk of chronic diseases [16]. Fruits and their related products have often regarded as important for their multifaceted role in improving health performance. Among fruits, the persimmon (*Diospyros kaki*), a deciduous fruit belonging to the Ebenaceae family, is widespread in Asian countries, particularly in China, Korea and Japan. It is cultivated world widely, with over 90% of its production occurring in East Asian countries. It has been used for years for various medicinal purposes, e.g., hypertension, treating coughs, frostbite, paralysis, bleeding and burns [17]. It has been previously reported that the persimmon is one of the most bioactive fruits, containing abundant bioactive compounds, for example phenolics, vitamin C and carotenoids [18]. Previous reports suggest that phenolic compounds such as gallic acid, tannin, proanthocyanidin, syringic acid, chlorogenic acid, catechin, epicatechingallate, sinapine, myricetin, and quercetin found in persimmon exhibit potent antioxidant, antidiabetic, anti-hyperlipidemic and neuroprotective activities [17,19]. Some studies have reported persimmon seeds to be a rich source of antioxidative compounds that can lower the risk of several fatal diseases caused by free radical mediated damage [20]. Jang et al. reported a stronger protective effect of persimmon seeds on oxidative DNA damages than other parts of persimmon [7]. Because of its medicinal as well as nutritional uses/values, persimmon may be considered a functional food.

In the current study, we aimed to analyze seeds from four different Korean persimmon cultivars for their in vitro effects against oxidative stress and determine their biochemical composition. In this regard, various functional biochemical analysis and quantifications of components such as free amino acids, organic acids and fatty acids were performed. Total phenolic and flavonoid content, antioxidant and acetylcholinesterase (AChE) inhibition activities were assayed for the methanolic extract of seeds of four different varieties of persimmons. The results of our study should help provide appropriate experimental evidence to employ the best seeds of these four Korean persimmon cultivars in the pharmaceutical, food and cosmetic industry.

## 2. Results and Discussion

### 2.1. Total Phenolic Content (TPC)

**Figure 1:**
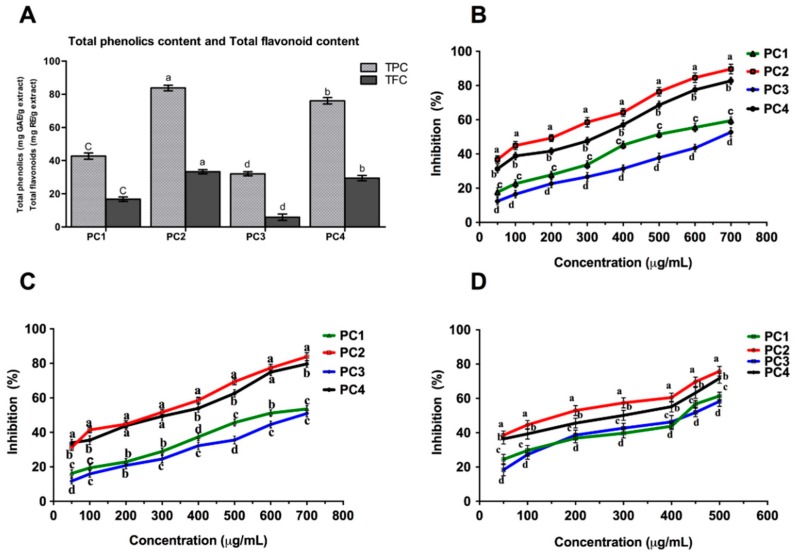
Total phenolics/ flavonoids and antixoidants activities of extracts of four persimmon cultivars. (**A**) Total phenolics and flavonoids content; (**B**) DPPH radical scavenging activity; (**C**) ABTS radical scavenging activity and (**D**) Superoxide radical scavenging activity of different persimmon cultivars seeds. Values in the different lower-case letters are significantly different at *p* < 0.05, (*n* = 3).

Phenolic compounds are the important class of secondary metabolites in plants that possess beneficial biological activity and may scavenge free radicals based on their hydrogen or electron donor ability. Phenolic compounds are considered vital in the food industry for improving food quality because of their ability to reduce lipid peroxidation [21]. Substantial variation was observed in total phenolic content among the different persimmon cultivars. The results showed that the total phenolic content of the persimmon seeds ranged between 32.04 ± 1.22 to 83.75 ± 1.72 mg∙GAE∙g^−1^ extract (Figure 1A).

The phenolic contents in the seeds of the persimmon cultivars were in the following order: PC2 > PC4 > PC1 > PC3. Cultivar PC2 had a significantly (*p* < 0.05) higher TPC, exhibiting almost 2-fold and 2.5-fold the phenolic content of PC1 and PC3, respectively. These findings for TPC were comparable to the results of previous studies on persimmon species. For instance, the phenolic contents were 42.3, 66.9, 15.20 mg∙GAE∙g^−1^ for *D. kaki* L. genotypes in Turkey, Japanese persimmon, and *D. kaki* var. silvestris M, respectively [22,23,24]. However, the TPC of our samples was lower than that of *D. kaki* L. cv. Daebong seed extract, which ranged from 386.25 to 440.29 mg∙GAE∙g^−1^ [20]. All four cultivars exhibited moderate to high amounts of phenolic compounds. A high TPC presence is considered beneficial for human health. The difference among the phenolic contents of the fruits of the analyzed persimmon cultivars might be attributed to the variation in geographical location, climatic and soil conditions, or pre-harvest factors. The TPC results from the persimmon seed extracts indicated they could be a good source of phenolic compounds that could be utilized both in the pharmaceutical and food industries.

### 2.2. Total Flavonoid Content (TFC)

Flavonoids are a class of polyphenolic compounds ubiquitously distributed in plants. They are considered beneficial for health as they have been shown to have antioxidative properties and inhibitory effects on carcinogenesis and mutagenesis in humans [24]. The flavonoid content of all cultivars was expressed as mg∙RE∙g^−1^ and shown in Figure 1A. The results varied from 5.86 ± 1.92 (PC3) to 33.28 ± 1.26 (PC2) mg∙RE∙g^−1^ of seed extract. The total flavonoid content in the seeds of the four cultivars was PC2 > PC4 > PC1 > PC3. Thus, PC3 had the lowest flavonoid content, which was approximately six times and five times less than that of cultivars PC2 and PC4, respectively. These results indicated that the trend for TFC was the same as that for TPC in the four cultivars. The TFC for all cultivars analyzed in the current study displayed similar results as those previously reported. Pu et al., [24] and Li et al. [25] who found 17.4 to 22.7 mg∙RE∙g^−1^ and 15.1 to 22.1 mg∙RE∙g^−1^ from the pulp of six *D. kaki* genotypes and tissues of astringent persimmons, respectively. The total flavonoid content of persimmon seed extract varied significantly among the four cultivars. This variation might have been caused by genetic differences between the cultivars, as genetically modified crops exhibit varying amounts of several functional metabolites [26]. The present study indicated that the flavonoids in extracts include a wide range of naturally occurring polyphenolic compounds in the persimmon seeds. In this regard, PC2 and PC4 cultivar seeds could be recommended as an excellent resource of bioactive compounds with significant benefits for the health and nutrition of consumers.

### 2.3. Oxidant Scavenging Potentials of Persimmon Seeds

#### Radical Scavenging Activity (DPPH, ABTS and SO Assays)

The DPPH radical scavenging assay is one of the most efficient and widely used methods for assessing the antioxidant potential of plant extracts. It is based on an electron-transfer reaction that produces a violet solution in ethanol. This free radical, stable at room temperature, is reduced in the presence of an antioxidant molecule, giving rise to a colorless ethanol solution [27]. The degree of reduction, in the form of discoloration, suggests the scavenging capacity of a tested sample. In our study, all the seed extracts exhibited DPPH radical scavenging potential. As Figure 1B demonstrates, the percentage of free-radical inhibition of seed extracts functioned in a dose-dependent manner (11.71% ± 1.6% to 83.84% ± 2.3%). EC_50_ values for all extracts with reference to the positive control, ascorbic acid, ranged from 706.37 to 262.65 µg∙g^−1^ (Table 1). The antioxidant activity of seed extracts on the DPPH radical was in the order PC2 > PC4 > PC1 > PC3. At the maximum concentration of 700 µg∙g^−1^, PC2 exhibited nearly 33%, 30%, and 14% more inhibition then PC3, PC1, and PC4, respectively. PC4 was the second top cultivar and exhibited 29% and 26% higher DPPH radical scavenging activity than that of PC3 and PC1, respectively. In comparison with the positive control, ascorbic acid, the scavenging effect of all extracts was lower. Based on our results, it is concluded that all cultivars, specifically PC2 and PC4, had strong DPPH radical scavenging potential by trapping its ions to form stable radicals and terminate radical chain reactions. The findings of the abilities of PC2 and PC4 are in agreement with previous studies [7,20,28] in which 88.98% and 83.37% inhibition was reported for ethanolic and methanolic extracts of L. cv. Daebong persimmon cultivar seeds, respectively. However, the DDPH radical scavenging activity reported in lyophilized persimmon pulp [22,23,24] was lower than that of the top cultivars (i.e., PC2 and PC4) at 700 µg∙g^−1^. The results indicated that all of the extracts, particularly PC2 and PC4, could be used as a potent source of natural antioxidants.

The ABTS method has been extensively used for the evaluation of antioxidant activity of foods [29]. The ABTS assay is mostly indicated for measurement of antioxidant activity of extracts containing hydrophilic compounds. This assay is based on determination of the discoloration in the ABTS assay solution when the antioxidants in the extract scavenge free radicals. As shown in Figure 1C, all four cultivar extracts exhibited moderate to strong concentration-dependent scavenging activities with PC2 being the cultivar that exhibited the highest ABTS radical scavenging activity at the maximum concentration, 700 µg∙g^−1^, which was statistically significantly greater (*p* < 0.05) than that of the other cultivars. However, the reference compound, ascorbic acid, exhibited higher activity than all of the extracts. The IC_50_ values of all samples ranged from 692.95 to 195.83 µg∙g^−1^, as presented in Table 1 and the ABTS radical scavenging activity of the extracts ranged from 12.36% to 89.66% inhibition and was ranked PC2 > PC4 > PC1 > PC3.

Superoxide (SO) anion is the most common radical produced in the body and it plays a vital role in the formation of reactive oxygen species such as singlet oxygen, hydrogen peroxide and hydroxyl radical, which cause oxidative damage to lipids, proteins and DNA [30]. We therfore aimed to assess the comparative potential of the sample extracts to counteract superoxide radical. In the current study, persimmon seed extracts showed potentials to inhibit superoxide radicals generated by a PMS/NADH system ranging from 18.36% ± 2.45% to 75.85% ± 2.8% in a concentration dependent manner (Figure 1D). Like the DPPH and ABTS assays, PC2 was noted to be the most prominent cultivar in scavenging superoxide anion radicals at the highest concentration, i.e., 500 µg∙g^−1^, which was closely followed by PC4 that exhibited 71.73% ± 2.7% inhibition. The SO scavenging activity of persimmon seeds extract was thus ranked in the following order: PC2 > PC4 > PC1 > PC3. PC3 displayed the lowest activity (58.29% ± 2.9%) at the maximum tested concentration, which was nearly 17% and 13% less than PC and PC4, respectively. The results were statistically significant (*p* < 0.05). The EC_50_ values of the persimmon seed extract against superoxide radical scavenging activity were found to be 408.75 to 190.27 µg∙g^−1^ as presented in Table 1. The EC_50_ values of PC2 and PC4 of persimmon seeds extract indicated slightly less activity than rutin (185.6 ± 4.1 µg∙g^−1^). The findings of this study were remarkably superior than the activity reported for Japanese persimmon pulp [23] at the maximum tested concentration, whereas, the activity reported for persimmon leaves in [31] was higher than that of the current persimmon cultivar seed extracts. The results indicate the persimmon seed extracts, particularly PC2 and PC4, are potent scavengers of superoxide radical like the standard rutin. Therfore, these Korean persimmon cultivar seed extracts could be used as easily available sources of natural antioxidants in the pharmaceutical and food supplement industries. However, further study on the isolation and characterization of the components responsible for the antioxidant activity of the extracts should be performed. PC2 had the highest levels of phenolic and flavonoid content, and displayed the strongest antioxidant activity, suggesting that phenols are a major contributing factor for the antioxidant activity of persimmon seed extracts. This finding is in agreement with those of previous reports [29,32] that indicated a positive correlation between antioxidant activity and phenolic content in fruits. In comparison with the DPPH assay results, relatively high scavenging activity was exhibited by extracts in the ABTS assay; this observation corroborates the findings of previous studies [29,33]. The ABTS assay has been recommended as the best assay for evaluating antioxidant potential in the persimmon [25]. The different antioxidant activities of the persimmon seed extracts could be due to the different structures of their respective components, which may influence the mechanism of their reactions with free radicals. Hence, small molecules that have high accessibility to the active site of the radical, might display high antioxidant activity [34]. The two-way ANOVA analysis showed that the different cultivars possessed significantly higher dose-dependent responses against oxidative stress as revealed by the ABTS, DPPH and SO tests (Table 2). Additionally, the acivity might be influenced by the presence in either lower or higher quantities of specific antioxidant agents such as phenols or flavonoids in the extracts, whilst, the actions of many other antioxidant compounds present in the samples, such as carotenoids, can contribute to the antioxidant activities as well [35]. Genotypic differences, and cultivation and environmental conditions might also influence the antioxidant capacity of fruits [36].

### 2.4. Acetylcholinesterase (AChE) Inhibition by Persimmon Seeds

**Figure 2:**
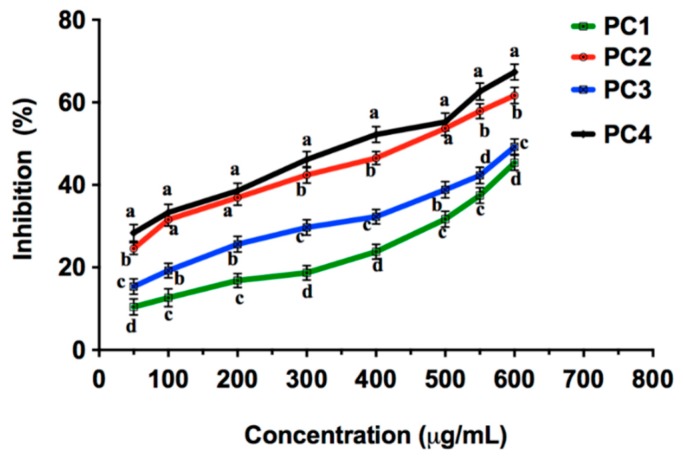
AChE inhibitory activity of different cultivars of persimmon seeds; Values in the different lower-case letters are significantly different at *p* < 0.05 (*n* = 3).

Research for beneficial phytochemical substances with low toxicity for AChE inhibition from natural resources has been of considerable interest. The methanolic extracts of the seeds of the four persimmon cultivars were comparatively assayed for AChE enzyme inhibition. Figure 2 depicts the dose-dependent effect of the extracts for AChE inhibition. PC4 and PC2 were the most effective AChE inhibitors at the highest tested concentration with 67.34% ± 1.89% and 61.65% ± 1.95% inhibition, respectively. The positive control, galantamine, showed remarkable AChE inhibition at very low concentrations, greater then all extracts (IC_50_ 0.78 ± 0.2 µg∙g^−1^). The IC_50_ values of methanolic extracts ranged from 842.19 to 390.37 µg∙g^−1^, as listed in Table 1. Several studies have demonstrated the AChE inhibitory role of various fruits. For example, Pervin et al. [37] reported 55.58% AChE inhibitory activity of grapes at 500 µg∙g^−1^, which is in agreement with the inhibitory activity of the PC3 and PC4 cultivars. Similarly, high molecular weight persimmon tannin has been shown to enhance memory performance in mice brains [38].

Because the PC4 and PC2 extract cultivars demonstrated the highest phenolic and flavonoid content, this may explain the high AChE inhibitory activity of these extracts. Previous studies have shown the AChE inhibitory potential of phenolic and flavonoid compounds [38,39]. Various phenolic compounds (i.e., rutin, tannin, and *p*-coumaric acid) have been reported in different cultivars of persimmon [24], which were confirmed as strong AChE inhibitors [40]. The two-way ANOVA analysis showed that the different cultivars possessed significantly higher dose-dependent AChE enzyme inhibition (Table 2). Phenolic compounds play a crucial role in protecting or delaying neurodegenerative diseases caused by different intracellular components, which may result in reduced chances of Alzheimer’s disease [41]. To best of our knowledge, there have been no reports on AChE inhibitory activity of persimmon seeds. making our study is the first to report the AChE inhibitory potential of persimmon seeds. Further studies are required to determine the bioactive components in the extracts responsible for AChE inhibition.

### 2.5. Free Amino Acid in Persimmon Seeds

Free amino acids present in plant foods are classified into two categories, essential and non-essential. The human body cannot produce essential amino acids; hence, they are required in adequate amounts in the diet. The composition of amino acids in all the cultivars is listed in Table 3. Statistical differences (*p* < 0.05) in the composition level of both essential and non-essential amino acids were observed.

The total amino acid amount was 2617.31 to 3773.05 μg∙g^−1^ dry weight. The PC4 cultivar possessed the highest level, followed by PC1, PC3, and PC2, respectively. The amounts of total amino acids in our study were in agreement with reports on different persimmon peels (2412.6–3714.5 µg∙g^−1^ dry weight) [42]. All of the analyzed cultivars exhibited a remarkable amount of essential amino acids, which ranged from 636.98 to 1557.69 µg∙g^−1^, which constituted approximately 22.76% to 41.28% of total free amino acids.

The PC4 seeds had the highest ratio (41.28%) of essential amino acid/total amino acid (EAA/TAA), followed by PC3 (40.24%), PC2 (29.24%), and PC1 (22.76%). According to the WHO and FAO, foods in the best protein category have an EAA/TAA ratio above 40% [43,44]. In our study, both PC4 and PC3 samples were close to or greater than 40%, suggesting they are good quality sources of amino acids.

All the studied samples contained modest to high levels of threonine, valine, methionine, isoleucine, leucine, phenylalanine, and lysine, although histidine was not detected in PC2 and PC4. Among the essential amino acids, valine and threonine were the most abundant amino acids in PC1 and PC2, whereas PC3 was rich in valine and leucine. However, threonine and leucine were the most abundant essential amino acids in the PC4 cultivar. Valine is considered essential for muscle development and coordination, and it is also a central nervous system stimulant. Threonine is required for the formation of the amino acids glycine and serine that aid in the synthesis of elastin, collagen, and muscle tissue. Threonine boosts the immune system and thus acts as an immuno-stimulant by promoting the growth of the thymus gland [45]. Leucine facilitates tissue, bone, and skin wound healing by modulating the natural pain reliever enkephalins [46] and also assists in regulating oxidative glucose utilization by skeletal muscles [47].

Among all free amino acids, glutamic acid was the distinct amino acid of the PC1 cultivar, whereas alanine appeared to be the main component of both PC2 and PC3, and aspartic acid was the most dominant component of PC4. Glutamic acid, alanine, and aspartic acids play beneficial roles in human health. Alanine has a remarkable role in maintaining balanced amounts of glucose and nitrogen in the human body via glucose alanine cycle [48]. From a nutritional perspective, aspartic acid is very important in higher plants, as it acts as a precursor for the synthesis of several essential amino acids [49], and functions as a excitatory neurotransmitter in the brain [50]. Glutamic acids are known for enhancing the taste of food, playing a role in brain metabolism [51], and are used for the treatment of various neurological disorders such as epilepsy [52].

Some of the following nitrogen-containing compounds were also detected in the persimmon seeds extracts: citrulline, β-alanine, γ-amino-*n*-butyric acid, citrulline, ethanolamine, ammonia, and 1-methylhistidine. These compounds are either synthesized post-translationally from other amino acids or produced independently in plants. Significant variations were observed in the sum of nitrogen-containing compounds in all four persimmon cultivars. Citrulline was the most abundant amino acid present in high amounts in all samples, ranging between 1777.15 ± 19 μg∙g^−1^ (PC1) and 2914.29 ± 14.7 μg∙g^−1^ (PC3), whereas the γ-amino-*n*-butyric acid compound level was the second highest in all cultivars and it is a vital neurotransmitter, contributing to behavioral functions of the brain [53]. However, β-alanine, ethanol amine, ammonia, and 1-methylhistidine levels were low in all cultivars and collectively constituted 12.1% to 14.9% of all nitrogen-containing compounds.

Overall, the PC4 cultivar was recognized as the best cultivar, followed by PC3, PC2, and PC1 when total free amino acids and N-containing compounds were taken into consideration. For food-derived products, free amino acid profiles could be a supportive parameter for quality assurance [54]. Because of distinctive profiles of free amino acids in the seeds of persimmon cultivars, PC4 and PC3 could be recommended for use as a quality control parameter for manufactured products.

### 2.6. Fatty Acids in the Seeds of Persimmon

The results of fatty acid profiles of persimmon seeds in the present study are provided in Table 4. The results indicated that the monounsaturated fatty acid oleic acid, and the polyunsaturated fatty acid linoleic acid were the most abundant in all the persimmon cultivars, and contributed 36.27% ± 1.1% to 43.22% ± 1.2% and 30.6% ± 0.88% to 39.63% ± 0.4% of total fatty acids, respectively. Palmitic acid, which constituted 17.54% ± 2.1% to 21.53% ± 1.08% (PC3 and PC2, respectively) of total fatty acids, was the third most important fatty acid. PC4 had the highest amount of oleic acid and lowest amount of linoleic acid. In contrast, PC3 showed a higher level of linoleic acid and lower level of oleic acid.

The ecological conditions and differences in cultivars could explain the variation in the level of fatty acids among persimmon cultivars. Overall, total analyzed fatty acid composition of persimmon seeds varied between 1180.5 µg∙g^−1^ (PC1) and 2503 µg∙g^−1^ (PC2), and was statistically significantly different (*p* < 0.05). Interestingly, the PC2 cultivar exhibited stronger antioxidant activities than other persimmon cultivars. Other studies [55] have made similar observations for *Centaurea* species. Hence, it might be hypothesized that for protection and maintenance of fatty acids, plants need to accumulate higher levels of antioxidants. In the previous studies, oleic acids and linoleic acids have been reported as the main fatty acids in persimmon fruits and seeds [7,56]. Oleic acid has been reported to act as an anti-inflammatory fatty acid by playing a major role in activating different immune competent cell pathways [57]. Linoleic acid is an essential fatty acid that has been described to have significant antioxidant activity [58]. Linoleic acid acts as the precursor for the entire omega-6 fatty acid family, and plays vital roles in reducing the incidence of cancer, autoimmune diseases, Alzheimer’s disease, and coronary heart disease [59]. The results of this comparative study of fatty acids in the four persimmon cultivars may aid in the development of cultivation practices for superior and nutrient-rich persimmon species.

### 2.7. Organic Acids Content in Different Persimmon Cultivar Seeds

Organic acid content is considered a vital factor of a fruit that contributes to the development of its taste. In persimmon seeds, oxalic, citric, malic, succinic, lactic, and acetic acid were identified among the organic acids (Table 5). All analyzed organic acids were detected in all the persimmon cultivars, excluding acetic acid, which was not identified in the PC1 and PC4 cultivars. Levels of total organic acids in the four persimmon cultivars ranged from 249.8 mg∙kg^−1^ DW in cultivar PC2 to 421.2 mg∙kg^−1^ in PC4. A statistically significant variation in terms of organic acids was found among all cultivars. Malic acid, as an individual organic acid, was abundant in all cultivars, with the highest concentration (3708 mg∙kg^−1^ dry weight) in PC4 cultivar and the lowest (1985 mg∙kg^−1^ DW) in PC2 cultivar. In [60] malic acid was identified as the main organic acid in various persimmon fruit cultivars, which is consistent with our results, although they reported a lower amount of malic acid (1044 to 401 mg∙kg^−1^ FW) than that in the present study. However, the content of malic acid in loquat seeds was higher (4620 mg∙kg^−1^ FW of malic acid) than that in our analyzed persimmon cultivar seeds [61]. These differences might possibly be caused by differences in climatic conditions, maturity stage, genotype of plants, and analytic procedures.

Citric acid, an important organic acid in food processing and used as an acidity regulator, was the second highest organic acid in all cultivars, ranging from 248 to 351 mg∙kg^−1^ DW. The content of citric acid reported by [61] in ripened persimmon fruits was the second most abundant organic acid, but measured in high quantities, which varied between 196 mg∙kg^−1^ to 701 mg∙kg^−1^. Analyzed persimmon seeds contained significantly lower amounts of citric acid compared with pawpaw and mayhaw seeds, in which citric acid levels were reported to be 2030 mg∙kg^−1^ and 4030 mg∙kg^−1^ FW, respectively [62]. Ripe tropical fruits, such as pineapple and papaya, have been reported to have much higher contents of citric acid [63]. Oxalic acid content was comparatively low and varied between 111 (PC1) and 193 mg∙kg^−1^ dry weight (PC4). Excessive amounts of oxalate in the form calcium and potassium oxalate are the common constituents of kidney stones. Hence, patients having a history of nephrolithiasis cannot be advised to consume fruits with high oxalic acid content [62]. All cultivars had succinic acid, lactic acid, and acetic acid in trace amounts, except PC1 and PC1in which acetic acid was not detected. Apart from influencing the flavor of fruits, these organic acids have also been proposed as a key to maintaining quality, ripeness, stability, and maturity in fruits [64].

### 2.8. Relationship between Total Phenolic/Flavonoid Content, Antioxidant Activities, and Acetyl-cholinesterase Inhibition

Several studies have described the correlation between total phenolic content and antioxidant activities of plants and reported a positive correlation [65]. The results of the present study demonstrate a strong correlation between total phenolic and flavonoid contents, and the antioxidant activities (DPPH and ABTS) of the four persimmon cultivar seeds. A strong correlation was found between TPC of persimmon seeds and % DPPH, % ABTS inhibition and superoxide anion scavenging activity, with R^2^ values of 0.985, 0.998, and 0.993, respectively. Likewise, an outstanding correlation appeared between total flavonoid content of seed extract and percent inhibition of DPPH , ABTS and superoxide radical with the R^2^ values of 0.928 and 0.970, respectively. These findings are in agreement with the reports of previous studies, which revealed the role of high phenolic content in boosting antioxidant activity [66]. Furthermore, a good linear correlation was observed between TPC, TFC of persimmon seed extracts, and acetylcholinesterase inhibition with R^2^ values of 0.720 and 0.606, respectively. This was in accordance with previously reports, which demonstrated the positive correlation of phenolic and flavonoid content with acetylcholinesterase inhibitory activity [67]. The findings of the current study suggest that the persimmon seed extracts contained bioactive phytoconstituents that may hold positive antioxidative and AChE inhibitory effects.

## 3. Materials and Methods

### 3.1. Chemicals

All chemicals, reagents, solvents, and standards used in the experiments were obtained from Sigma Chemical Co. (St. Louis, MO, USA). Doubled distilled deionized water (Milli-Q 18.2 MΩ·cm^−1^, 25 °C, Millipore, Billerica, MA, USA) was used in all experiment. All chemicals were of analytical grade.

### 3.2. Plant Materials

Fruits seeds of four different persimmon cultivars, Sangju Doongsi, Gyoungsan Ban si, Jinan Gal gam and Sang gam doongsi (hereafter referred to as PC1, PC2, PC3 and PC4, respectively) obtained from the Sangju Persimmon Experiment Station (Sangju, Korea), were harvested at “ready-to-eat” maturity stage in October 2014. The cultivars were grown under different geographic and climatic conditions. Seeds were washed with tap water to remove any adhering pulp and kept for drying in hot air drying oven at 50 °C for 72 h. Dried seeds were ground into a fine powder (60-mesh size) with a FM-681C blender (Hanil, Gwangju, Korea), packed in airtight plastic bags and stored at 4 °C for future analysis.

### 3.3. Preparation of Extracts of Persimmon Seeds

The air and shade-dried persimmon seed samples (30 g) were extracted by mixing them into 300 mL of 80% methanol at 25 °C at 150 rpm for 24 h and filtering through filter paper. The residue was then extracted with two additional treatments with 300 mL of methanol, as described above. The combined 900 mL 80% methanol extracts were then rotary evaporated at 40 °C to dryness and kept in the dark at 4 °C for further analysis.

### 3.4. Determination of Total Phenolic Content

The total phenolic constituent of the methanolic extract was determined using the method of [68], which employed a Folin-Ciocalteu reagent and gallic acid as standard. Briefly, 0.1 mL of extract solution (1 mg/mL) of persimmon seeds and 0.1 mL of Folin-Ciocalteu reagent were added and mixed thoroughly. After 3 min, 1 mL of 2% Na_2_CO_3_ was added, and then the mixture was allowed to stand for 40 min at room temperature for color development. The absorbance was measured at 750 nm using a spectrophotometer (Cat. Log. No. 51119300, ThermoFischer Scientific, Espoo, Finland). The standard calibration curve was plotted using gallic acid. The total phenolic content of the seeds from the four persimmon cultivars (PC1, PC2, PC3, and PC4) was expressed as gallic acid equivalents in mg∙g^−1^ of extract.

### 3.5. Determination of Total Flavonoid Content

Total flavonoid content was measured using the modified colorimetric method described in [68]. Briefly, 400 µL of persimmon seed extract solution (1 mg/mL) was appropriately diluted and mixed with 1 mL of 5% sodium nitrite solution and reacted for 6 min followed by the addition of 0.15 mL of 10% aluminum chloride. After 5 min, 0.4 mL of 1 M sodium hydroxide solution was added. Finally, 0.4 mL of distilled water was added and the absorbance was measured immediately at 510 nm using a model spectrophotometer (Thermo Fischer Scientific, Espoo, Finland). The flavonoid content was calculated from a rutin standard curve and expressed as rutin equivalents (RE, mg/g extract).

### 3.6. DPPH Radical-Scavenging Activity

The antioxidant activity (AA) of persimmon seed extract was assayed based on the scavenging activities of the stable 2,2-diphenyl-1-picrylhydrazyl free radical, as described by [69], with slight modifications. Briefly, a 0.1 mM solution of DPPH in 100% methanol was prepared. Subsequently, 4 mL of the sample solution in water at different concentrations (50 µg∙g^−1^ to 700 µg∙g^−1^) was added to 1 mL of this solution. The solution was shaken vigorously and kept in the dark at room temperature for 30 min until stable absorption values were obtained. The absorbance was measured at 517 nm using a spectrophotometer (ThermoFischer Scientific). Ascorbic acid was used as the control. The DPPH radical scavenging activity was calculated by the following equation:

Scavenging effect (%) = 1 – (Abs_sample_ − Abs_control_) × 100 (1)

### 3.7. ABTS Radical-Scavenging Activity (RSA)

The spectrophotometric assay of ABTS radical scavenging activity was measured as described by [70], with slight modifications. Briefly, the ABTS cation radical was generated by reacting 7 mM ABTS solution with 2.45 mM potassium persulfate, which was allowed to stand in the dark at room temperature for 14 h. Before usage, the solution was diluted to achieve an absorbance of 0.700 ± 0.025 at 734 nm with 95% ethanol. To determine the scavenging activity, the ABTS reagent was mixed with the sample at different concentration (50 µg∙g^−1^ to 700 µg∙g^−1^) or negative control (95% ethanol) (0.3 mL) and the absorbance at 734 nm was measured 6 min after the initial mixing, using 95% ethanol as the blank. Ascorbic acid was used as a positive control. The scavenging capability of the ABTS radical was calculated using the following equation:

Inhibition % = 1 – (Abs_sample_ − Abs_control_) × 100 (2)

### 3.8. Superoxide Radical-Scavenging Activity

The superoxide radical scavenging activity of persimmon seeds extract was measured according to the previously described method by Aydin [71] with the following modifications: superoxide radicals were generated in the PMS-NADH system containing 4 mL Tris-HCl buffer (16 mM, pH 8.0), 348 µM NADH (adenine dinucleotide), 80 µM NBT (nitroblue tetrazolium), and 30 µM PMS (phenazine methosulphate). Different concentrations of samples ranging from 50 to 500 µg/mL were added to the to the PMS-NADH system for free radical scavenging. The reaction mixture was incubated at ambient temperature for 5 minutes and then absorbance was measured at 562 nm against the blank. In the control, the sample was replaced with Tris-HCl buffer. All tests were performed 4 times. Rutin was used as positive control. The ability of persimmon seed extract to scavenge superoxide radical was calculated using the following equation:

Scavenging effect % = (1 − A_Sample562_/A_control562_) × 100 (3)

### 3.9. Micro-Plate Assay for Inhibition of Acetylcholinesterase

Inhibition of acetylcholinesterase (AChE) activity was measured using Ellman’s colorimetric method, with slight modifications, as described by Moyo et al. [72]. Briefly, the following buffers were prepared and used for the assay: Buffer A (50 mM Tris-HCl, pH 8.0), Buffer B (50 mM Tris-HCl, pH 8.0, containing 0.1% bovine serum albumin-BSA), Buffer C (50 mM Tris-HCl, pH 8.0, containing 0.1 M NaCl, 0.02 M MgCl_2_∙6H_2_O). In a 96-well plate, 25 µL of 15 mM acetylthiocholine iodide (ATCI) in water and 125 µL of 3 mM 5,5-dithiobis-2-nitrobenzoic acid (DTNB) in buffer C were added separately. Subsequently, 50 µL of Buffer B and 25 µL of plant extracts with different concentration (50 µg∙g^−1^ to 600 µg∙g^−1^) were added in sequence. Absorbance was measured spectrophotometrically at 412 nm, three consecutive times with 45-s intervals. Thereafter, AChE (25 µL, 0.2 U∙mL^−1^) was added and absorbance read at every 45 s (five times). Galantamine (0.5–5 μg∙mL^−1^) was used as a positive control. The percentage of AChE inhibition was calculated using this following equation:

Inhibition % = 1 – (sample reaction rate/blank reaction rate) × 100(4)

### 3.10. Free Amino Acid Composition

Free amino acid composition of persimmon cultivars was analyzed as described by [73]. Briefly, approximately 500 mg of ground sample from each cultivar was hydrolyzed in 5 mL of 6 N HCl under a vacuum in an ampulla tube for 24 h at 110 °C. The suspension was then filtered and evaporated under a vacuum. The solid residue was dissolved in 2 mL of deionized water and evaporated twice. The final residue was dissolved in 10 mL of 0.01 N HCl and filtered through a 0.45 μm filter membrane using an automatic amino acid analyzer (L-8900 Hitachi, Tokyo, Japan). An amino acid standard mixture solution (type H) for automatic amino acid analysis was purchased from Wako Pure Chemical Industries, Ltd. (Osaka, Japan) and used for the accurate analysis of amino acids composition. All of the samples were run in triplicate and expressed in µg∙g^−1^ of dry weight.

### 3.11. Fatty Acids Contents Analysis

Fatty acid content of persimmon seeds was analyzed by the method described in [74]. The ground powdered seeds (1 g) of persimmon from each variety were extracted with *n*-hexane (10 mL). The samples were then placed in a shaking incubator (150 rpm) at 50 °C for 2 days. The supernatant was separated by centrifugation (1200× *g* at 25 °C) followed by transfer into new tubes. Hexane was removed by using a continuous flow of nitrogen. The extracted material from each sample was placed in a screw-capped vial, and 5 mL of methylation solution (H_2_SO_4_/methanol/toluene 01:20:10 mL) was added. The sealed vial was heated in a water bath (100 °C) for 60 min and allowed to cool to room temperature. Then, 5 mL of water was added and the mixture was shaken. The mixture was separated into two layers, and the upper layer was removed using a Pasteur pipette and dried using anhydrous sodium sulfate for 5 min. GC-MS analysis was conducted with the Agilent Model 7890A series system (Agilent, Dover, DE, USA) equipped with an Agilent 5975C MS detector and an Agilent 7683 autosampler, and an MS ChemStation A.03.00 was used. The GC-MS was equipped with a DB-5MS capillary column (30 m × 0.25 mm i.d. × 0.25-μm film thickness; J & W Scientific-Agilent, Folsom, CA, USA), whereas helium was used as a carrier gas with a flow rate of 0.6 mL·min^−1^ in split mode (1:50). A 1-µL sample was directly injected into the GC using an automatic sampler (Agilent 7683B). The injector and detector temperatures were 120 and 200 °C, respectively. The column temperature was programmed from 50 to 200 °C at 10 °C·min^−1^ and then held at 200 °C for 5 min. The mass conditions were as follows: ionization voltage, 70 eV; scan rate, 1.6 scan∙s^−1^; mass range, 30–450; and ion source temperature, 180 °C. The components were identified based on their relative retention time and mass spectra compared with standards, Wiley7N, NIST library data of the GC-MS system, and data from the literature.

### 3.12. Organic Acid Composition

Quantification of organic acid was determined using the method described in Waqas and co-authors [75]. The HPLC analysis for quantification of organic acids was performed with a Waters (Millford, MA, USA) model 600E system equipped with a Refractive Index detector (RI, model 410). One milliliter of ultrapure water was used for every milligram of organic acids to prepare standards of pure organic acids as well as standard mixture of all acids for calibration curves at various concentrations. Samples were prepared by extracting air dried grounded samples in distilled water. The samples were flushed with nitrogen and centrifuged for 15 min. One mL of the sample (0.1 g) was added to 9 mL of distilled water and left overnight at room temperature. Then the sample was passed through a 0.22-µm syringe filter (Millipore, Billerica, MA, USA) and injected (20 µL). The isocratic conditions for HPLC analysis were as follows: mobile phase: 0.005 M H_2_SO_4_ in water, flow rate 0.6 mL/min, column temperature 65 °C (PL Hi-Plex H column (7.7 mm × 300 mm) specially designed for organic acids analysis).

### 3.13. Statistical Analysis

The data were analyzed statistically for standard deviation and error by using GraphPad Prism (Ver 5.0; San Diego, CA, USA). The mean values were compared using Duncan’s multiple range tests at (*p* < 0.05) employing the statistical software program SAS (version 9.2, Cary, NC, USA). GraphPad Prism was used for graphical presentations.

## 4. Conclusions

The present study reports a detailed evaluation of the in vitro antioxidant activity, acetylcholinesterase inhibition, total phenolic/ flavonoid content, and nutritional composition, which included total essential amino acid, fatty acid, and organic acid content, of four Korean persimmon cultivar seed extracts. The results suggest that persimmon seeds are a novel and interesting natural source of bioactive human health-promoting compounds. Among the studied varieties, PC2 and PC4 proved to be the most significant in antioxidant activity, acetylcholinesterase inhibition, total phenolic content, and flavonoid content. A positive correlation was established between phenolic content of the seed extract and antioxidant and AChE inhibitory activity. Furthermore, all of the cultivars showed remarkable nutritional compositions, including higher levels of essential fatty acids, amino acids, and substantial organic acid content levels. The differences among the persimmon cultivars might have occurred because of genetic differences in cultivars, soil conditions, and climatic conditions. The reported variation may also be considered valuable for selecting desirable persimmon genotypes with ideal phytonutrient composition for commercial cultivation. The promising nutritional composition and antioxidative and AChE inhibitory activities suggest that persimmon seeds, which are a underutilized and neglected part of the fruit, are a novel health-promoting resource, which could be of keen interest for health specialists and in the food industry. As an active source of natural antioxidants and acetylcholinesterase inhibitors, further study on persimmon seeds should be carried out for identification and isolation of bioactive constituents, determination of the molecular mechanisms responsible for the antioxidant activity, and assessing their effectiveness by in vivo techniques.

## Figures and Tables

**Table 1:** IC_50_ (µg/g) values of different persimmon cultivar seeds extract and positive control.

Samples	DPPH Radical Scavenging	ABTS Radical Scavenging	AChE Inhibition	SO Radical Scavenging
PC1	609.03 ± 4.11	517.74 ± 3.37	779.16 ± 4.19	396.34 ± 3.97
PC2	262.65 ± 2.04	195.83 ± 1.95	427.68 ± 3.78	190.27 ± 2.96
PC3	706.37 ± 4.06	692.95 ± 4.17	659.96 ± 4.06	408.75 ± 3.72
PC4	297.55 ± 2.42	285.66 ± 2.73	368.29 ± 2.68	260.51 ± 4.1
Ascorbic acid	112.23 ± 2.51	101.41 ± 1.96		
Galantamine			0.78 ± 0.2	
Rutin				185.63 ± 3.62

**Table 2:** Two-way ANOVA of the bioassays performed for different cultivars of persimmon seeds and their concentrations.

Assays	Concentrations of Seed Extracts			Types of Persimmon Seeds			Interaction of Concentration *x* Types		
	MS	P	% of Total Variance	MS	P	% of Total Variance	MS	P	% of Total Variance
DPPH inhibition	3040	0.0001	59.16%	4657	0.0001	38.84%	21.76	0.0001	1.27%
ABTS inhibition	3419	0.0001	58.42%	5346	0.0001	39.15%	30.35	0.0001	1.56%
SO inhibition	2095	0.0001	73.00%	1335	0.0001	23.26%	10.81	0.1971	1.13%
AChE inhibition	1919	0.0001	59.74%	2865	0.0001	38.22%	11.15	0.0002	1.04%

MS = mean of squares; P = *p* value; % of total variance suggest whether the effects are modulated by the kind of variety vs. difference in concentrations. The increasing % show significantly higher results. Analysis were performed using determined by two-way ANOVA followed by a Bonferroni *post-hoc* test with a *p* < 0.05 for concentration, cultivar and their interaction (GraphPad prism 6.01; La Jolla, CA, USA).

**Table 3:** Amino acid analysis of the four different cultivars of persimmon seeds.

Amino Acids	PC1	PC2	PC3	PC4
Threonine	129.97 ± 2.6 ^d^	141.18 ± 1.7 ^c^	167.03 ± 3.6 ^b^	289.078 ± 2.64 ^a^
Valine	140.51 ± 3.4 ^d^	186.62 ± 3 ^c^	228.35 ± 3.21 ^b^	269.18 ± 3.3 ^a^
Methionine	36.32 ± 4.3 ^d^	44.6 ± 2.6 ^c^	93.45 ± 2 ^b^	127.63 ± 1.2 ^a^
Isoleucine	98.85 ± 1.1 ^d^	134.41 ± 2.7 ^c^	142.04 ± 2.5 ^b^	251.11 ± 2 ^a^
Leucine	94.48 ± 2.16 ^d^	123.59 ± 2 ^c^	218.31 ± 3.5 ^b^	293.87 ± 2 ^d^
Phenylalanine	66.04 ± 4.5 ^d^	74.24 ± 1.1 ^c^	119.13 ± 0.9 ^b^	168.9 ± 3 ^a^
Lysine	52.28 ± 1.2 ^d^	60.67 ± 4.6 ^c^	102.46 ± 2.2 ^b^	157.9 ± 4 ^a^
Histidine	18.49 ± 1.3 ^a^	ND ^c^	14.59 ± 3.8 ^b^	ND ^c^
Aspartic acid	292.98 ± 3.6 ^b^	275.4 ± 3.01 ^c^	253.26 ± 1.7 ^d^	662.81 ± 1.1 ^a^
Alanine	458.46 ± 1.3 ^b^	522.44 ± 5.1 ^a^	436.25 ± 1.6 ^c^	304.55 ± 2.2 ^d^
Cystine	24.14 ± 1.09 ^d^	64.53 ± 3.1 ^b^	73.93 ± 4.3 ^a^	42.17 ± 2.01 ^c^
Glutamc acid	932.74 ± 1.2 ^a^	444.02 ± 3 ^b^	264.24 ± 4.4 ^d^	369.56 ± 2.0 ^c^
Glycine	51.31 ± 3.2 ^c^	139.61 ± 1.7 ^a^	126.54 ± 2.2 ^b^	140.77 ± 4.03 ^a^
Sarcosine	101.44 ± 4.2 ^a^	55.96 ± 3.0 ^b^	101.17 ± 2.1 ^a^	84.94 ± 2.1 ^c^
Tyrosine	94.39 ± 2.9 ^d^	118.34 ± 1.05 ^c^	148.29 ± 2.01 ^b^	194.21 ± 1.6 ^a^
Serine	205.95 ± 3.01 ^c^	231.66 ± 2.03 ^b^	207.79 ± 2.2 ^c^	416.29 ± 3.1 ^a^
Arginine	ND ^a^	ND ^a^	ND ^a^	ND ^a^
Citrulline	1777.15 ± 19.1 ^d^	2205.1 ± 15.6 ^c^	2914.29 ± 14.7 ^a^	2454.09 ± 12.4 ^b^
β-Alanine	42.89 ± 2.05 ^c^	55.34 ± 2.2 ^b^	76.81 ± 3.4 ^a^	53.47 ± 3.1 ^b^
γ-Amino-*n*-butyric acid	174.81 ± 7.77 ^c^	620.44 ± 11.1 ^a^	599.41 ± 12.87 ^ab^	577.91 ± 18.01 ^b^
Ethanolamine	44.65 ± 3.3 ^b^	65.05 ± 4.8 ^a^	63.81 ± 2.0 ^a^	45.4 ± 4.1 ^b^
Ammonia	26.64 ± 1.2 ^bc^	28.7 ± 2.2 ^b^	24.52 ± 1.04 ^c^	33.01 ± 2.9 ^a^
1-Methylhistidine	174.88 ± 3.1 ^d^	254.58 ± 1.4 ^c^	322.48 ± 3.2 ^b^	399.01 ± 3.02 ^a^
Total A. A	2798.35	2617.31	2696.91	3773.05
Total essential A. A	636.98	765.34	1085.4	1557.69
Total Metabolites	2241.0005	3229.21	4001.33	3562.89

Average ± standard error from three separate replicates. Values with different lower-case letters in the same line are significantly different at *p* < 0.05 as determined by Duncan’s test. Total essential amino acids = sum of Histidine, Isoleucine, Leucine, Lysine, Methionine, Phenylalanine, Threonine, and Valine. Total = sum of 17 amino acids. Total metabolites = sum of Citrulline, β-Alanine, γ-Amino-n-butyric acid, Ethanolamine, Ammonia, 1-methylhistidine.

**Table 4:** Fatty acid composition of persimmon cultivar seeds, expressed as % of total fatty acids.

Cultivars	Palmitic	Stearic	Oleic	Linoleic	Linolenic	Arachidic	Total µg/g
	C16:0	C18:0	C18:1	C18:2	C18:3	C20:0	
PC1	17.67 ± 1.17 ^b^	3.26 ± 0.34 ^a^	37.51 ± 0.91 ^b^	38.05 ± 1.02 ^b^	3.49 ± 1.01 ^b^	ND	1180.5 ^d^
PC2	21.53 ± 1.08 ^b^	3.46 ± 0.81 ^a^	38.82 ± 1.02 ^b^	33.21 ± 0.72 ^c^	2.97 ± 0.69 ^b^	ND	2503.5 ^a^
PC3	17.54 ± 2.1 ^b^	3.57 ± 0.78 ^a^	36.27 ± 1.1 ^bc^	39.63 ± 0.4 ^a^	2.97 ± 0.98 ^b^	ND	1701.25 ^c^
PC4	19.95 ± 1.9 ^ab^	3.46 ± 1.2 ^a^	43.22 ± 1.2 ^a^	30.60 ± 0.88 ^d^	2.76 ± 1.1 ^b^	ND	1851.1 ^b^

**N**D = not detected. Average ± standard error from three separate replicates. Values with different lower-case letters in the column are significantly different at *p* < 0.05 as determined by Duncan’s multiple range test.

**Table 5:** Organic acids composition of persimmon seeds, expressed in (mg/kg dry weight).

Cultivars	Oxalic Acid	Citric Acid	Malic Acid	Succinic Acid	Lactic Acid	Acetic Acid	Total
PC1	111 ± 1.0 ^d^	248 ± 1.9 ^d^	3510 ± 15.1 ^b^	46 ± 1.7 ^a^	23 ± 0.7 ^a^	ND ^c^	3938 ^c^
PC2	144 ± 2.0 ^c^	307 ± 4.2 ^b^	1985 ± 4.3 ^d^	21 ± 0.8 ^c^	18 ± 1.0 ^b^	23 ± 1.8 ^a^	2498 ^d^
PC3	178 ± 2.4 ^b^	351 ± 4.3 ^a^	3263 ± 8.9 ^c^	21 ± 1.2 ^c^	11 ± 0.8 ^c^	15 ± 1.0 ^b^	3839 ^b^
PC4	193 ± 3.1 ^a^	270 ± 3.4 ^c^	3708 ± 6.1 ^a^	35 ± 0.9 ^b^	6 ± 0.5 ^d^	ND ^c^	4212 ^a^

ND = not detected. Average ± standard error from three separate replicates. Values with different lower-case letters in the same column are significantly different at *p* ≤ 0.05 as determined by Duncan’s multiple range test.
